# Supplementary material for: Milk levels of transforming growth factor beta 1 identify mothers with low milk supply
Source: PLoS One. 2024 Jun 13;19(6):e0305421. doi: 10.1371/journal.pone.0305421 (PMC11175467; doi:10.1371/journal.pone.0305421)
Supplement: Attachment — Submitted filename: Response to reviewers.docx [file pone.0305421.s002.docx]

We thank the reviewers for their time and expertise. We have incorporated each of their recommendations into our revised manuscript, and we feel this has greatly strengthened the paper. Below is a point-by-point response to each reviewer.

**Reviewer #1:**

Line 43; please add reference.

We have added two references supporting this statement.

Line 131; please fix the TGF□1 and TGF□2

We have corrected the TGF beta nomenclature throughout the text and figures.

Discussion: A gap remains in the discussion regarding the feasibility and cost-effectiveness of implementing TGF measurement. Addressing this gap would strengthen the overall impact and translatability of the research.

Thank you for this excellent suggestion. We’ve added text to the discussion about the feasibility and cost-effectiveness of TGF measurement. This should add transparency about the strengths and limitations of this approach.

**Reviewer #2:**

The study demonstrates great execution and writing, addressing a clinically significant topic supported by thorough bench research. The researchers have adhered to rigorous and comprehensive methodologies, with meticulous attention given to controls, including control vehicles and the assessment of miRNA expression differentials. Certain enhancements could further improve the comprehensiveness of the study:

- Rectifying typing errors on line 131.

These errors have been corrected.

- Incorporating a table, if feasible, to outline maternal characteristics to enrich clinical correlation.

We have added Table 1, describing maternal, infant, and milk factors.

- Including a reference validating the Dietary Screener Questionnaire (DSQ).

This reference has been added.

- Clarifying in the methods section that milk samples were collected only once from each mother, rather than “at each study visit” as stated in line 122 to improve the accuracy of the study's methodology.

We apologize for this oversight. The revised manuscript clarifies that only one sample was analyzed for each mother.

- Providing additional details regarding the number of milk samples analyzed (139 vs. 27) to derive the relationship patterns for TGF β1 measurements in Figure 2c and 2d to enhance transparency and interpretation of the findings. Was there any difference in patterns between mothers with and without LMS?

Thank you for this suggestion. The legend for Figure 2 now includes the number of samples used to derive the relationships for TGFβ1. All of the mothers in the LMS group (27/27) and non-LMS group (112/112) provided a sample for analysis. The levels of TGFβ1 were higher in mothers with LMS (U = 1070, p = 0.009).

- Correction of reference 32 in line 278. This article does not attend to guilt and frustration in mothers with lactation difficulties. It is a strong point. May consider under mentioned references instead.

Jackson L, De Pascalis L, Harrold J, Fallon V. Guilt, shame, and postpartum infant feeding outcomes: A systematic review. Matern Child Nutr. 2021;17(3):e13141. doi:10.1111/mcn.13141

Kam RL, Bennetts SK, Cullinane M, Amir LH. "I didn't want to let go of the dream": Exploring women's personal stories of how their low milk supply was discovered. Sex Reprod Healthc. Published online February 11, 2024. doi:10.1016/j.srhc.2024.100953

We apologize that our reference list was misaligned with the text in our original submission. We have corrected this error and added the two references above.

**Reviewer #3**:

This is a well written, interesting piece of work. As a neonatologist working in a breastfeeding friendly NICU, I find it extremely important to begin to have an understanding of why some mothers struggle to produce enough breast milk to feed their babies. Further study may find that TGF B1 is a useful marker to identify these mothers early in the post-partum period.

Thank you. We agree that this is an important field of study!
